# The impact of forced displacement: trauma, increased levels of inflammation and early presentation of diabetes in women Syrian refugees

**DOI:** 10.1093/pubmed/fdad037

**Published:** 2023-04-05

**Authors:** Thenmozhi Venkatachalam, Siobhán O'Sullivan, Daniel E Platt, Walid Ammar, Randa Hamadeh, Naji Riachi, Diane Presley, Brigitte Khoury, Dominique Gauguier, Moni Nader, Lu Qi, Pierre Zalloua

**Affiliations:** College of Medicine and Health Sciences, Khalifa University, Abu Dhabi, UAE; College of Medicine and Health Sciences, Khalifa University, Abu Dhabi, UAE; Computational Biology Center, IBM TJ Watson Research Centre, Yorktown Heights, NY, USA; Faculté de Médecine, Université Saint Joseph, Beirut, Lebanon; PHC Department, Lebanese Ministry of Public Health, Global Team of Experts (GHTE), Beirut, Lebanon; College of Medicine and Health Sciences, Khalifa University, Abu Dhabi, UAE; College of Medicine and Health Sciences, Khalifa University, Abu Dhabi, UAE; Department of Psychiatry, American University of Beirut, Beirut, Lebanon; Université de Paris, INSERM UMR 1124, Paris 75006, France; College of Medicine and Health Sciences, Khalifa University, Abu Dhabi, UAE; Biotechnology Center, Khalifa University of Science and Technology, Abu Dhabi, UAE; Department of Epidemiology, School of Public Health and Tropical Medicine, Tulane University, New Orleans, LA, USA; College of Medicine and Health Sciences, Khalifa University, Abu Dhabi, UAE; Biotechnology Center, Khalifa University of Science and Technology, Abu Dhabi, UAE; Harvard TH Chan School of Public Health, Boston, MA, USA

**Keywords:** diabetes, forced displacement, inflammation, refugee, stress

## Abstract

**Background:**

Forced displacement and war trauma cause high rates of post-traumatic stress, anxiety disorders and depression in refugee populations. We investigated the impact of forced displacement on mental health status, gender, presentation of type 2 diabetes (T2D) and associated inflammatory markers among Syrian refugees in Lebanon.

**Methods:**

Mental health status was assessed using the Harvard Trauma Questionnaire (HTQ) and the Hopkins Symptom Checklist-25 (HSCL-25). Additional metabolic and inflammatory markers were analyzed.

**Results:**

Although symptomatic stress scores were observed in both men and women, women consistently displayed higher symptomatic anxiety/depression scores with the HSCL-25 (2.13 ± 0.58 versus 1.95 ± 0.63). With the HTQ, however, only women aged 35–55 years displayed symptomatic post-traumatic stress disorder (PTSD) scores (2.18 ± 0.43). Furthermore, a significantly higher prevalence of obesity, prediabetes and undiagnosed T2D were observed in women participants (23.43, 14.91 and 15.18%, respectively). Significantly high levels of the inflammatory marker serum amyloid A were observed in women (11.90 ± 11.27 versus 9.28 ± 6.93, *P* = 0.036).

**Conclusions:**

Symptomatic PTSD, anxiety/depression coupled with higher levels of inflammatory marker and T2D were found in refugee women aged between 35 and 55 years favoring the strong need for psychosocial therapeutic interventions in moderating stress-related immune dysfunction and development of diabetes in this subset of female Syrian refugees.

## Introduction

Refugees experience high rates of post-traumatic stress disorder (PTSD), anxiety and depression from forced displacement and civilian war trauma. As of January 2020, it is estimated that ~5.5 million Syrian refugees have fled conflict and violence and settled predominantly in the Middle East and North Africa region, mainly in Turkey, Lebanon, Jordan and Iraq, outside of camp settings in both urban and semi-urban areas.^1^ Lebanon has the highest per capita concentration of Syrian refugees in the world with an estimated 183 refugees per 1000 inhabitants (UNHCR 2015).

Studies have shown that refugees endure high levels of acute stress and adverse lifestyle changes due to their forced displacement.^2^ Changes in diet, social isolation and exclusion, limited access to healthcare, separation from family, stress, anxiety and trauma associated with violence have been associated with the occurrence of chronic non-communicable diseases such as type 2 diabetes (T2D).^3^ In addition, clinically meaningful reductions in PTSD symptoms are associated with a lower risk of T2D.^4^ Furthermore, forced displacement contributes to psychosocial stress and vulnerability; both of which are directly associated with increases in serum blood glucose levels and T2D. Moreover, PTSD has been shown to alter the production and secretion of pro-inflammatory cytokines and acute phase proteins [e.g. C-reactive protein (CRP) and serum amyloid A (SAA)], which stimulate an immune response through the induction of NF-κB.^5^^,^^6^ Chronic activation of these pathways over periods of time can result in physiological responses becoming sensitized, dysfunctional and maladaptive. Previous research on the Syrian refugee populations in Lebanon and neighboring countries has demonstrated a high prevalence of T2D among the adult refugees.^7^

Pro-inflammatory cytokines have also been shown to be elevated in individuals exposed to trauma and different forms of abuse compared with non-exposed healthy individuals.^8^ Coupled to the pro-inflammatory cytokine response, levels of acute phase proteins such as CRP and SAA can also change during periods of trauma and infection.^9^^,^^10^ Increased levels of CRP have been reported in individuals with generalized anxiety disorder. Although conflicting studies report no relationship between PTSD and CRP, they have highlighted the potential role of demographic factors such as race and sex in determining the response to trauma.^11^ Gender differences in pro-inflammatory response have been reported and experimentally induced inflammation was more significantly associated with socioemotional changes in women compared with men.^12^

The correlation between trauma-induced PTSD, depression anxiety and production of inflammatory markers has not been always confirmed and even contradictory in the literature due to differences in study design features, including meaningful demographic factors such as race, ethnicity and sex.^6^^,^^13–15^ The comprehensive data collected for this study from the Syrian refugee population in Lebanon present a unique opportunity to explore factors that may be involved in the development of T2D, due to the trauma of forced displacement. In this study, we investigate the relationship between stress, inflammation, psychopathology, development of T2D and obesity in trauma-exposed Syrian refugees.

## Methods

The findings presented here are derived from the first set of data collected from a pilot cohort of 303 Syrian refugees, recruited from primary healthcare centers (PHCs), Lebanon between June 2019 and March 2020. Details on ethical approvals, study population, data collection and clinical assessment, mental health assessment, variables definition and statistical analysis are provided in the supplementary materials. The mental health of the recruited subjects was assessed using the Harvard Trauma Questionnaire (HTQ) and the Hopkins Symptom Check List-25 (HSCL-25) questionnaires. The HTQ and HSCL are the diagnostic tools for PTSD and anxiety/depression, respectively.

## Results

### Demographic structure of the study population

This study included participants above 18 years old attending a PHC in Lebanon. The average age of the study participant was 47 years, and the study population was composed of 32.3% men and 67.6% of women. A large number or participants were from Aleppo (48.84%), Idlib (8.25%) and Raqqa (6.6%) where most of the fighting occurred (Fig. S1**)**. The average age of men and women participants was 50.57 ± 12.52 and 44.28 ± 12.89, respectively. The average body mass index (BMI) of the study participants was 29.38 kg.m^2^ with women displaying a substantially higher BMI (30.81 ± 6.69 kg.m^2^) than their men counterparts (27.94 ± 4. 69 kg.m^2^) with *P* value < 0.0001. About 55.57% study participants fell in the category of overweight and obese, whereas 4.95% of women participants were extremely obese.

Patient demographic and familial history of metabolic diseases are summarized in Table 1. The majority of the study participants (73.93%) were < 55 years old and 90.33% were married. Most participants self-reported familial metabolic disease such as T2D (59.74%), hyperlipidemia (26.40%) and hypertension (58.08%). Approximately half of the study participants over 55 years old, irrespective of gender, had a family history of T2D or hypertension (59.74 and 58.08%).

**Table 1:** Demographic and familial history of metabolic diseases among Syrian refugees

	Men (*N* = 98, 32.3%)	Women (*N* = 205, 67.6%)	Total (303)	*P* value
	Mean ± STD	Mean ± STD		
**Age (Mean, SD)**	50.57 ± 12.52	44.28 ± 12.89	47.42 ± 12.71	0.000077^*^
**Weight**	81.25 ± 14.22	77.39 ± 16.1	79.32 ± 15.16	0.0437^*^
**Height**	157.55 ± 47.03	149.46 ± 35.96	153.50 ± 41.49	0.099
**BMI**	27.94 ± 4.69	30.81 ± 6.69	29.38 ± 5.69	0.000162^*^
	**Total (Percentage)**	**Total (Percentage)**	**Total (Percentage)**	
**Age group**				
<55	61 (20.13)	163 (53.8)	224 (73.93)	0.0017^*^
>55	36 (11.88)	40 (13.2)	76 (25.08)	
**Marital status**				
Single	8 (2.64)	10 (3.30)	18 (5.94)	0.3022
Married	84(27.72)	180 (59.41)	264 (87.13)	
Divorced	-	5 (1.65)	5 (1.65)	
Widow	-	5 (1.65)	5 (1.65)	
**Body mass index**				
Healthy	25 (8.25)	36 (11.88)	61 (20.13)	0.9673
Overweight	34 (11.22)	42 (13.86)	76 (25.08)	
Obese	28 (9.24)	71 (23.43)	99 (32.67)	
Extreme obese		15 (4.95)	15 (4.95)	
**Smoking history**				
Non-smoking	23 (7.59)	127 (41.9)	150 (49.5)	<0.0001^*^
Smoking	56 (18.48)	65 (21.45)	121 (39.93)	
Ex-smoking	15 (4.95)	6 (1.98)	21 (6.93)	
**Family history of metabolic diseases**				
**T2D**				
Diabetic	60 (19.80)	121 (39.93)	181 (59.74)	0.6114
ND	35 (11.55)	82 (27.06)	117 (38.61)	
**Hyperlipidemia**				
Yes	28 (9.24)	52 (17.16)	80 (26.40)	0.4822
No	63 (20.79)	143 (47.19)	206 (67.98)	
**Hypertension**				
Yes	50 (16.50)	126 (41.58)	176 (58.08)	0.1283
No	44 (14.52)	75 (24.75)	119 (39.27)	

Chi-square test was performed for the categorical variables and the *t*-test was performed for the mean values. Categorical variables were displayed as counts and percentages.

No significant difference in the prevalence of T2D and prediabetes was observed between men and women participants (Table S1). Although no difference was seen in the fasting blood sugar (FBG) and HbA1c levels, it is observed to be increased with age in both genders. Significantly high levels of uric acid and creatinine were observed in men (5.33 ± 1.75 and 0.89 ± 0.62 mg/dl) with *P* values of 0.0033 and < 0.000001 (Table 2).

**Table 2:** Clinical characteristics of the study population aged <35 (between 35 and 55 years) and >55 and assessment of trauma/anxiety

	Men				Women				*P* value
	<35 (*n* = 9) Mean ± SD	35–55 (*n* = 52) Mean ± SD	>55 (*n* = 36) Mean ± SD	Total (*n* = 97) Mean ± SD	<35 (*n* = 61) Mean ± SD	35–55 (*n* = 102) Mean ± SD	>55 (*n* = 40) Mean ± SD	Total (*N* = 203) Mean ± SD	
Total cholesterol	181.4 ± 38.18	174.4 ± 42.52	162.17 ± 47.25	172.66 ± 42.65	175.56 ± 34.75	195.25 ± 42.81	190.63 ± 65.86	187.14 ± 47.81	0.011611^*^
HDL	39.4 ± 8.26	38.98 ± 10.69	40.54 ± 9.42	39.64 ± 9.46	52.28 ± 15.48	47.79 ± 12.98	47.97 ± 16.22	49.35 ± 14.90	<0.000001^*^
LDL	115.9 ± 37.67	106.02 ± 46.26	97.85 ± 47.02	106.59 ± 43.65	98.26 ± 23.48	118.43 ± 35.59	111.29 ± 62.49	109.33 ± 40.52	0.594201
Triglycerides (TG)	238.6 ± 159	170.26 ± 42.52	132.87 ± 60.24	180.58 ± 112.08	116.34 ± 76.70	161.32 ± 135.84	157.1 ± 94.37	144.92 ± 102.30	0.006572^*^
FBG	119.6 ± 46.48	141.4 ± 117.01	146.21 ± 64.22	135.74 ± 55.47	116.61 ± 59.02	128.88 ± 55.40	163.52 ± 92.96	136.34 ± 69.13	0.940943
HbA1c	6.4 ± 2.72	6.68 ± 55.69	7.03 ± 1.86	6.7 ± 2.14	5.81 ± 1.45	6.38 ± 1.76	7.05 ± 1.58	6.41 ± 1.60	0.194478
Uric acid	5.2 ± 1.8	5.46 ± 1.77	5.33 ± 1.69	5.33 ± 1.75	4.28 ± 0.92	4.50 ± 2.07	5.51 ± 1.32	4.77 ± 1.44	0.003358^*^
Creatinine	0.78 ± 0.23	0.82 ± 0.21	1.07 ± 1.42	0.89 ± 0.62	0.62 ± 0.13	0.62 ± 0.15	0.67 ± 0.19	0.64 ± 0.15	<0.000001^*^
WBC	7.06 ± 1.41	8.053 ± 1.52	7.9 ± 1.74	7.67 ± 1.56	7.28 ± 1.67	7.49 ± 1.55	11.72 ± 18.47	8.83 ± 7.23	0.119464
Hemoglobin (Hb)	16.07 ± 2.08	15.30 ± 1.21	14.42 ± 1.50	15.27 ± 1.60	13.00 ± 1.27	12.59 ± 1.69	12.93 ± 1.56	12.84 ± 1.51	<0.000001^*^
Hematocrit (HCT)	45.36 ± 4.98	43.38 ± 3.07	43.21 ± 9.10	43.98 ± 5.71	37.82 ± 3.22	37.47 ± 6.04	38.42 ± 4.03	37.9 ± 4.43	<0.000001^*^
Platelet (Plt)	209.25 ± 65.01	253.23 ± 59.42	231.79 ± 74.16	231.42 ± 66.19	265.36 ± 73.09	276.61 ± 76.51	270.56 ± 85.63	270.84 ± 78.41	0.000026^*^
CRP	1.21 ± 1.57	0.47 ± 0.59	0.48 ± 0.97	0.72 ± 1.05	0.49 ± 0.61	0.80 ± 0.94	0.91 ± 1.40	0.73 ± 0.98	0.905447
SAA	11.67 ± 7.90	8.66 ± 9.4	7.52 ± 3.51	9.28 ± 6.93	9.86 ± 10.27	12.04 ± 8.47	13.81 ± 15.05	11.90 ± 11.27	0.03602^*^
HTQ	1.81 ± 0.45	1.96 ± 0.51	1.88 ± 0.59	1.88 ± 0.52	2.03 ± 0.42	2.18 ± 0.43	1.91 ± 0.43	2.04 ± 0.43	0.004856^*^
HSCL-25	1.79 ± 0.48	2.01 ± 0.63	2.06 ± 0.77	1.95 ± 0.63	2.13 ± 0.51	2.26 ± 0.53	2.02 ± 0.46	2.13 ± 0.50	0.006232^*^

Multiple ‘*t*’-test was performed between men and women groups. *P* < 0.05 was considered significant.

Women had significantly higher total cholesterol (187.14 ±  47.81 mg/dl) and high-density lipoprotein (HDL) (49.35  ± 14.90 mg/dl) with *P* values of0.011 and < 0.0001 (Table 2). Men participants had substantially greater triglyceride levels (180.58 ± 112.08) with *P* value of 0.0065. No gender difference in the levels of low-density lipoprotein (LDL) was observed in the study cohort.

### Inflammatory markers

Higher CRP levels were observed in women in the age groups of 35–55 and > 55 years (0.80 ± 0.94 and 0.91 ± 1.40). However, no significant difference in the levels of CRP was observed in men (0.72 ± 1.05) and women (0.73 ± 0.98) participants (Table 2). In contrast, SAA levels were found to be significantly high in women participants (11.90 ± 11.27) with *P* value of 0.036. Furthermore, SAA levels were significantly correlated with gender (*P* = 0.027), BMI (*P* = 0.003) and CRP levels (*P* < 0.0001) (Table S2).

### Mental health assessment

Higher prevalence of symptomatic PTSD with HTQ was observed in women aged 35–55 years (56/102, 54.90%) followed by women aged <35 years (21/61, 34.42%). Similarly, higher prevalence of symptomatic anxiety/depression score was observed in women aged 35–55 years (74/102, 72.54%) followed by women aged <35 years (37/61, 60.65%). With HSCL-25, both men and women had elevated anxiety/depression scores, regardless of age and gender (Table 2). However, women displayed remarkably higher symptomatic anxiety/depression scores (2.13 ± 0.50, *P* = 0.00623), and this effect was consistently observed in the three age groups. Remarkably, highest score was observed in the age group of 35–55 (2.26 ± 0.53). On the other hand, highest anxiety/depression score in men was observed in the age group of > 55 years (2.06 ± 0.77). Similar trend was observed with HTQ, where only women participants aged between 35 and 55 years had symptomatic anxiety/depression score higher than 2.06 (2.18 ± 0.43). Even though, women displayed higher symptomatic anxiety/depression score than men (2.04 ± 0.43 versus 1.88 ± 0.52, *P* = 0.0048), no symptomatic PTSD scores with HTQ were seen in men participants across all the age groups.

### Diabetic categories

Based on the FBG and HbA1c levels, three groups were established including non-diabetic (ND) (*n* = 115), not previously diagnosed diabetic (NPDD) (*n* = 65) and previously diagnosed diabetic (PDD) (*n* = 110) **(**Table 3**)**. Higher prevalence of diagnosed diabetes was observed in women (21.12%) than men (14.85%). Similarly, a greater than 2-fold increase in the prevalence of NPDD was observed in women (15.18%) compared with men (6.27%). Among the PDD group, the mean ages were 54.71 ± 12.35 and 52.48 ± 11.46 years for men and women, respectively. Among the NPDD group, the mean ages were 49.18 ± 10.56 and 45.77 ± 8.85 years for men and women, respectively (Table 3).

**Table 3:** Clinical characteristics of the diabetes subgroups in the study population

		Age	Cholesterol	HDL	LDL	TG	FBG	HbA1c	CRP	SAA	HTQ	HSCL-25
**Previously diagnosed diabetic**	Men 45, 14.84%	54.71 ± 12.35	155.36 ± 45.09	40.19 ± 10.42	93.19 ± 51.60	145.57 ± 95.42	172.03 ± 67.65	8.06 ± 2.07	0.64 ± 0.45	8.19 ± 9.05	1.92 ± 0.54	2.02 ± 0.71
	Women 64, 21.12%	52.48 ± 11.46	184.95 ± 52.08	44.14 ± 13.01	101.6 ± 38.68	198.24 ± 158.26	184.23 ± 86.86	7.79 ± 1.78	0.59 ± 0.96	11.11 ± 8.36	2.01 ± 0.42	2.09 ± 0.44
		0.334	0.0026^*^	0.094	0.33	0.049^*^	0.432	0.4677	0.745	0.0855	0.33	0.526
**Not previously diagnosed diabetic**	Men 19, 6.27%	49.18 ± 10.56	183.02 ± 44.25	39.35 ± 9.24	114.5 ± 46.81	159.65 ± 104.39	150.06 ± 24.81	6.84 ± .61	0.37 ± 0.12	9.46 ± 4.21	2.2 ± 0.69	2.12 ± .83
	Women 46, 15.18%	45.77 ± 8.85	193.45 ± 50.11	47.04 ± 11.13	114.49 ± 40.37	170.94 ± 84.71	147.99 ± 25.67	7.28 ± 1.62	1.09 ± 1.29	13.38 ± 9.04	2.25 ± 0.41	2.38 ± 0.54
		0.221	0.408	0.0055^*^	0.999	0.677	0.762	0.114	0.0003^*^	0.019^*^	0.769	0.213
**ND**	Men 31, 10.23%	45.65 ± 12.19	180.5 ± 36.49	39.13 ± 8.24	109.49 ± 33.75	168.71 ± 104.57	100.38 ± 14.84	5.28 ± 0.27	0.29 ± 0.16	7.96 ± 4.39	1.76 ± 0.39	1.83 ± 0.55
	Women 84, 27.72%	36.55 ± 10.69	189.05 ± 36.49	53 ± 15.59	114.12 ± 30.96	114.70 ± 58.58	97.022 ± 16.93	5.28 ± 0.18	0.41 ± 0.32	10.97 ± 9.59	2.14 ± 0.46	2.26 ± 0.55
		0.00016^*^	0.267	0.0000007^*^	0.4889	0.000690^*^	0.332	>0.999	0.048^*^	0.095	0.000082^*^	0.000312^*^

Multiple ‘*t*’-test was performed between total men and women groups. *P* < 0.05 was considered significant. (^*^) indicate statistical significance.

PDD women presented with higher levels of cholesterol and triglyceride (184.95 ± 52.08 mg/dl and 198.24 ±  158.26 mg/dl) than men (155.36 ± 45.09 mg/dl and 145.57 ± 95.42 mg/dl) (*P* = 0.0026 and 0.049). Inflammatory markers of CRP and SAA were found to be significantly higher in NPDD women (1.09 ± 1.29 and 13.38 ± 9.04) than men (0.37 ± 0.12 and 9.46 ± 4.21) with *P* values of 0.0003 and 0.019, respectively (Table 3).

ND women (controls) displayed higher HDL cholesterol levels (53 ± 15.59 mg/dl, *P* = < 0.0001) and men displayed higher triglyceride levels (168.71 ± 104.57 mg/dl, *P* = 0.00069) (Table 3). Interestingly, higher levels (marginal in the case of CRP) of inflammatory markers were observed in NPDD women (CRP: 1.09 ± 1.29 and SAA: 13.38 ± 9.04) than men (CRP: 0.37 ± 0.12 and SAA 9.46 ± 4.21) with *P* values of 0.0003 and 0.019, respectively. Furthermore, BMI and CRP levels strongly correlated with the diagnosis of T2D (main outcome variable) in the study population (Table S3).

### Predictive model of SAA and CRP with stress scores for T2D in refugee population

To identify predictive trends for the onset of T2D progression in the refugee population, we carried out receiver operating characteristic analysis. Among the inflammatory markers tested, SAA levels exhibited a significantly high predictive value of 63.7% (*P* < 0.0001) followed by the CRP levels of 60.8% (*P* = 0.0040) (Fig. S2). Similarly, the predictive trend employed for the anxiety/depression and HSCL-25 predicted T2D onset with 59.7% (*P* = 0.012) followed by HTQ (57.8%) with *P* value of 0.0040.

**Fig. 1:**
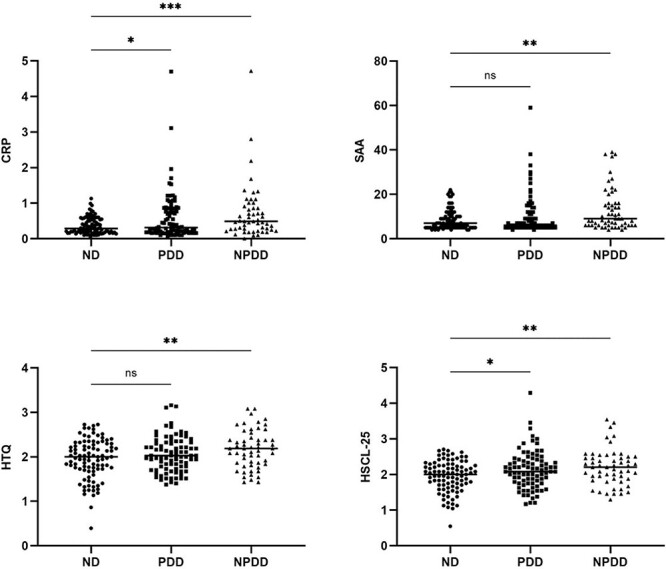
Levels of inflammatory markers (SAA and CRP) and PTSD (HTQ and HSCL-25) among various diabetes categories. ND = non-diabetes; PDD = previously diagnosed diabetes; NPDD = not previously diagnosed diabetes. One-way ANOVA with multiple comparison using Tukey’s multiple comparisons test. ^*^^*^P < 0.01, ^*^^*^^*^^*^P < 0.0001; ns = not significant.

The levels of the inflammatory markers were investigated across all study groups: ND, PDD and NPDD (Fig. 1). The CRP levels were significantly higher in the PDD and NPDD groups, whereas SAA levels were significantly high in the NPDD group when compared with the ND. The SAA levels did not vary between the ND controls and PDD groups. The Hopkins anxiety/depression scores were significantly high in the PDD and NPDD groups when compared with the ND control group. The HTQ score, however, was significantly higher in the NPDD group compared with the ND. We sought to test whether the effects of trauma (PTSD), anxiety and depression, measured by the Harvard and Hopkins scores, impacted the onset of diabetes and obesity.

The regressions predicted T2D according to the anxiety/depression scores without (Table S4) and with (Table S5) adjustment by age, hypertension and hyperlipidemia. The results of these regressions are also presented in Fig. S3. Within the estimated sampling variation, the two anxiety/depression scores did not yield significant associations. Regressions analysis predicting T2D according to the anxiety/depression scores with adjustment by sex are shown in Table S6 and Fig. S3c and d. The results were suggestive, but not significant, given the estimated sampling variation ranges. The *P* values of multivariate logistic regression model showed variations within expected ranges for sampling variations.

## Discussion

### Main finding of this study

We report a link between the inflammatory profile, gender and the development of T2D in a refugee population. Young Syrian refugee women, especially between the ages of 35 and 55 years exhibit more chronic PTSD trajectories and higher levels of inflammatory and anxiety/depression markers than men. The elevated levels of acute phase proteins of CRP and SAA are suggested to be the potential indicators of trauma-related pathophysiology and systemic inflammation. These markers have been linked to an increased risk, and later development, of diabetes among women experiencing war trauma. This is one of the unique studies that explicitly investigates the link between the inflammatory profile, gender and the development of T2D in a traumatized population.

#### The gender effects

The gender is an important risk factor for the development of trauma-related disorders among subjects exposed to specific forms of potentially traumatic events.^16^ Women refugees report higher levels of depression, anxiety and somatization than trauma-affected refugee men.^17–21^ Although both men and women in our study had symptomatic anxiety/depression scores with HSCL-25, only women had considerably higher mean anxiety/depression scores and this was most apparent in the age group of 35–55. Gender differences in the proinflammatory cytokine response after severe trauma may account for the development of trauma-related psychopathologies^22^ and suggest that gender is a biological variable that moderates the relationship between inflammation and psychological symptoms.^12^ Subclinical inflammation mediated by body fat has been shown to affect CRP levels more strongly in women than in men.^23^ Accordingly, women participants showed higher prevalence of overweight than men, which accounts for the lower CRP levels seen in men. Also, the differences in the number of study participants may have, in part, influenced the gender effects observed. The number of male subjects in the study population was considerably less than the female subjects and this difference may have had an impact on gender effect observed. Anxiety and depression are powerful players in the regulation and dysregulation of the hypothalamic–pituitary–adrenal (HPA) axis and its links to other cerebral circuits. The HPA axis, the autonomic nervous system and the immune system work together in harmonizing the hormonal inflammatory stress response.^24^^,^^25^ Sex-specific epigenetic and neuroendocrine influences and immune responses contribute to differential responses to trauma. The females show increased glucocorticoid production in response to various acute stressors supported by the findings of sex differences at all levels of the HPA axis. The impact of anxiety/depression on women can be magnified by at least two factors: leaving their primary home under duress and caring for their children while displaced. Women have been reported to have a larger cortisol response to stress and lower proinflammatory cytokines, which impact the stress response chain linked with the HPA axis and are associated with greater PTSD risk.

#### Obesity

Trauma is high among Syrian refugees^26^^,^^27^ and several studies have shown that physiological stress that arises from the occurrence of traumatic life changing events also increases the risk of obesity and have adverse effects on the body’s physiological functions.^28^ It has been reported that the prevalence of obesity is higher among immigrant women than men and men being more likely to be overweight than immigrant women.^29–31^ In line with previous reports, we found a higher prevalence of obesity in women and higher prevalence of overweight in men. Several traumatic factors have been identified as a risk factor for increased obesity in refugee women, but the mechanism by which obesity regulated stress remains unclear.^32^^,^^33^

#### Inflammation

Numerous findings indicate a link between PTSD, depression and anxiety with inflammation.^34^ The psychological response to stress causes various endocrine perturbations, which lead to obesity, inflammation and insulin resistance.^35^^,^^36^ Inflammation appears to be higher in adults exposed to early life adverse events.^35^^,^^36^ In particular, elevated levels of acute phase proteins such as CRP and SAA have been associated with trauma-related pathophysiology as levels of these proteins have been found to increase during infection and trauma.^11^ In this study, refugee women had significantly higher levels of inflammatory marker SAA. Although CRP levels in women with PTSD are not always correlated, it has been reported that SAA levels correlate with PTSD symptoms in adolescent girls and women.^37^^,^^38^ Our results confirm that the effect of stress on inflammation is greater in traumatized women than men. As with the HTQ and HSCL-25 scores, the levels of both CRP and SAA are most elevated in women aged 35–55 years.

#### Inflammation and T2D

Large-scale genetic and epidemiological studies have underlined the prominent role of environmental factors in T2D etiology.^39^ High prevalence of T2D was observed in the refugee population studied. This prevalence is significantly higher than that in the general Syrian population^40^ as well as in the Lebanese population^41^ where the Syrian refugee population under study is currently living. Likewise, a nearly 2.5-fold increase in the prevalence of pre-diabetes was observed in the Syrian refugee population when compared with the general Syrian population and the host Lebanese population.^42^ Interestingly, women displayed significantly higher prevalence of early diabetes at a very young age than men. These results suggest that women are more prone to physiological stress mediated by obesity and inflammation, which is likely to increase the risk of developing T2D at an early age.

The inflammatory markers of SAA and CRP analyzed in our study, while generally on the lower levels considering the relatively young age (mean age 47.4) of the study population, are found to be increased in patients with T2D.^43^^,^^44^ The elevated levels of CRP are linked to increased HbA1c and are a risk factor for the development of diabetes and insulin resistance.^44–46^ Similarly, higher levels of SAA have been reported in obese subjects and have been shown in mice to be a marker of insulin resistance and to regulate insulin sensitivity when treated with recombinant SAA.^47–49^ Furthermore, our findings of higher SAA and CRP levels in NPDD and PD women are consistent with the results from prospective studies that have reported associations between depressive symptoms and T2D in women.^50^ Although we cannot rule out the role of poor nutrition and other socioeconomics factors, increased prevalence of T2D in refugees in our study, measurements of inflammatory markers (SAA and CRP) and the results from the trauma and anxiety and/depression questionnaires provide confirmatory evidence of relationships between the psychological stress and T2D risk^51^ and suggest their potential impact in the development of T2D in the study population.

### Limitations of this study

Psychopathology was determined in our study from self-reported questionnaires rather than a clinical interview, which would have given more details on the trauma experienced post displacement. However, two independent self-reported trauma questionnaires were administered in an adapted Arabic form to suit the study subjects. A longitudinal study would have been measured for the levels of inflammatory markers and glycemic control over time and would have allowed for repeated tests of recent T2D and pre-diabetes in the study sample, which together with sample sizes limits the study’s ability to isolate emergent and prodromal T2D cases using blood markers. Furthermore, determining the undiagnosed diabetes before displacement was challenging, and hence they were not considered as cases. Our study is further limited with the lack of control groups attending the same PHCs. Notwithstanding the limitations of the study, the uniqueness of our findings in this small population provides a significant contribution to the understanding of the relationship between trauma-related disorders, gender and inflammation. Our findings are critical and show that there is an urgent need not only to alleviate the symptoms of traumatic stress disorder but also to prevent further deterioration in the health of the refugees. If trauma leads to inflammation and subsequent development of early diabetes and obesity in young women, then early-stage therapeutic interventions for PTSD, anxiety and depression may moderate immune dysfunction through improving stress-related immune damage.

## What this study adds

This study is the first to document association between anxiety/depression (or trauma-like), inflammation, obesity and T2D in young Syrian refugee women especially between 35 and 55 years. Anxiety/depression-induced obesity and inflammation pose a significant T2D risk in Syrian refugees in Lebanon. Although more research is needed to identify the pathways that may explain the link between anxiety/depression, inflammation and T2D, psychosocial interventions aimed at mitigating the effects of stressful life changes may aid in the reduction of obesity and the prevention of T2D at an early age.

## Conflict of Interest statement

None declared

## Funding

PZ and LQ acknowledge the support from NIH, grant number 1R21TW010790-01A1.

## Authors’ contributions

Conceptualization: PZ, LQ, BK. Methodology: TV, WA, RH, BK, MN. Data Analysis: TV, DEP, NR, PZ. Manuscript drafting: TV, SS, DG. Manuscript review and editing: DEP, NR, DP, DG, BK, MN, LQ, PZ. Funding acquisition: PZ, LQ.

## Data availability

All data are presented in the manuscript.

## Supplementary Material

Supplementary_file_R1_Clean_fdad037

## Notes

**Thenmozhi Venkatachalam**, PhD

**Siobhán O'Sullivan**, PhD

**Daniel E. Platt**, PhD

**Walid Ammar**, MD

**Randa Hamadeh**, MD

**Naji Riachi**, MD

**Diane Presley**, MSc

**Brigitte Khoury**, PhD

**Dominique Gauguier**, PhD

**Moni Nader**, PhD

**Lu Qi**, MD/PhD

**Pierre Zalloua**, PhD
