# Supplementary material for: The impact of forced displacement: trauma, increased levels of inflammation and early presentation of diabetes in women Syrian refugees
Source: J Public Health (Oxf). 2023 Apr 5;45(3):e437–46. doi: 10.1093/pubmed/fdad037 (PMC10470347; doi:10.1093/pubmed/fdad037)
Supplement: Supplementary_file_R1_Clean_fdad037 [file supplementary_file_r1_clean_fdad037.docx]

**The Impact of forced displacement: Trauma, Increased levels of inflammation and Early Presentation of Diabetes in Women Syrian Refugees.**

**Supplementary file**

**Methodology**

**Study Population**

The findings presented here are derived from a pilot cohort of 303 Syrian refugees, sequentially recruited from primary healthcare centers (PHCs) serving Lebanese nationals and Syrian refugee patients in Lebanon between June 2019 to March 2020. Ethical approval for this study was granted by the institutional review board and ethics committee of the Lebanese American University in Beirut Lebanon and was conducted in compliance with the Declaration of Helsinki of 1975, revised in 2013. Informed consent written in the refugees’ native language (Arabic) was explained and signed by participants before being recruited for the study. Patients who agreed to take part in the study were asked to answer a series of questions (section IV of the Harvard Trauma Questionnaire, HTQ, the Hopkins Symptom Checklist-25, HSCL-25) and blood and plasma samples were collected for further biochemical analysis. Collected Blood and plasma samples transported to the laboratory at +4°C.

**Data Collection and clinical assessment**

Demographic and baseline clinical data for the participants were collected from the centralized electronic medical records systems of the PHC. This data included: socio-demographic characteristics (age), gender, nationality, place of residence, clinical characteristics such as previously diagnosed diseases (diabetes, CVD, hyperlipidemia, hypertension) and anthropometric measurements: height (cm), weight (kgs) and BMI (kg/m2).

Duplicates of blood and plasma samples were collected by venipuncture at the clinics from overnight fasting patients. Blood and plasma samples collected were analyzed at one designated clinical laboratory for basic laboratory parameters of blood homeostasis, kidney and liver function. These included: hemoglobin A1c (HbA1C, %), fasting blood glucose, total cholesterol (mmol/l), triglycerides (mmol/l), high-density lipoprotein (HDL, mmol/l), low-density lipoprotein (LDL, mmol/l), and creatinine (mmol/l) (nmol/l). Levels of inflammatory markers, SAA and CRP levels were also determined using immuno-phelometry on a BNII analyzer using an N Latex-enhanced test (Siemens, Schwalbach, Germany). The intraassay coefficient of variation was < 7% for duplicate tests for both CRP and SAA. CRP levels in the serum was determined using Eurolyser CRP assay kit (Eurolyser Diagnostica GmbH, AUSTRIA). The duplicate samples collected were stored on ice and transferred to our research laboratory for plasma and serum separation, DNA extraction and subsequent storage at -80°C.

**Mental health assessment**

Impact of war-trauma, migration, and psychological disorders in refugee’s mental health namely PTSD, anxiety, and depression was examined using two reliable and validated assessment tools that measure the impact of war trauma and its psychological sequelae: Hopkins Symptom Check List 25 (HSCL-25)(1, 2) and, Harvard Trauma Questionnaire (HTQ)(3, 4). HTQ has been reformulated, adapted, and copiously refined to make it congruent with DSM 5 criteria for PTSD and is considered theoretically informed and “refugee friendly” and refugee focused across many cultures (5). In HTQ, the first 16 symptoms from section IV were used to identify patients with PTSD(6). As with similar refugee populations studied, the threshold of >2.06 was used to identify cases with PTSD in this refugee sample from non-cases(7). The HSCL-25 consists of 10 anxiety and 15 depression questions. An average item score greater than 1.75 was used as a diagnostic proxy of anxiety and depression with good reliability and validity in clinical refugee samples (8). The pre-war status of each participant in the study was determined and patients with a psychiatric history were excluded from the cohort to avoid confounding variables. Furthermore, both HSCL-25 and HTQ scales have been frequently used among refugees and in population-based surveys and have been demonstrated to have sound psychometric properties among Arabic speakers, the native language of our study subjects (7, 9).

**Variable definitions**

The status of T2D was determined using measurements of fasting blood glucose, HbA1c, and self-reported information. T2D status was based on the CDC criteria as follows: FBG ≤99 mg/dL is considered normal, 100 to 125 mg/dL prediabetic, and ≥126 mg/dL specified diabetes; HbA1c, <5.7% is considered normal, 5.7%- 6.4% prediabetic and ≥ 6.5% indicates diabetes.

Participants who had previously been informed of having diabetes or were on diabetes medication upon enrolment in the study were assigned to the Previously Diagnosed Diabetic (PDD) group.

Subjects with fasting blood glucose between 100-125mg/dL or HbA1c between 5.7%-6.4% were classified as pre-diabetic. Since the number of newly diagnosed cases of diabetes was very small (those with >125mg/dL or >6.5%), the pre-diabetes and newly diagnosed diabetes groups were collectively referred to as Not Previously Diagnosed Diabetics (NPDD). The non-diabetics were designated ND.

Based on the CDC criteria, a BMI of (18.5-24. 9kg.m^-2^) was considered normal, (25-29. 9kg.m^-2^) considered overweight, (30-34. 9kg.m^-2^) considered obese and BMI ≥ 35kg.m^-2^ considered extremely obese. Total cholesterol levels >500 mg/dL were considered very high, and triglycerides of 150-199 mg/dL were rated borderline high.

**Statistical analysis**

Categorical variables were displayed as counts and percentages. Normally distributed continuous data were given as mean ± standard deviation (SD). In the univariate analysis, continuous data were analyzed by a Student t-test while the categorical data was compared using χ^2^test. Pearson’s rank correlation was used to assess the association between SAA, HSCL-25, and HTQ scores. CRP, SAA, Hopkins and Harvard scores were used to create ROC curves. An AUC’s of 0.7-0.8 was considered good; 0.6-0.7 was considered average and <0.6 was considered poor. GraphPad Prism (V8.1.2) was used to do the statistical analysis, which included the t-test and one-way ANOVA. Differences with P<0.05 were considered statistically significant.

We also applied logistic regression as implemented in the Statsmodels package(10) in python3 to compute the impact of stress measured by the Harvard and Hopkins scores on refugees. The logistic regression was performed for Non-diabetic and Diabetic (PDD, NPDD) with and without inclusion in the regression of hypertension, hyperlipidemia, and age, and regressions stratified by sex for the augmented list.

**References**

1. Hollifield M, Warner TD, Jenkins J, Sinclair-Lian N, Krakow B, Eckert V, et al. Assessing war trauma in refugees: properties of the Comprehensive Trauma Inventory-104. J Trauma Stress. 2006; 19:527-40.

2. Mahfoud Z, Kobeissi L, Peters TJ, Araya R, Ghantous Z, Khoury B. The Arabic validation of the Hopkins Symptoms Checklist-25 against MINI in a disadvantaged suburb of Beirut, Lebanon. The International Journal of Educational and Psychological Assessment. 2013; 13:17-33.

3. Shoeb M, Weinstein H, Mollica R. The Harvard trauma questionnaire: adapting a cross-cultural instrument for measuring torture, trauma and posttraumatic stress disorder in Iraqi refugees. The International journal of social psychiatry. 2007; 53:447-63.

4. Arnetz BB, Broadbridge CL, Jamil H, Lumley MA, Pole N, Barkho E, et al. Specific trauma subtypes improve the predictive validity of the Harvard Trauma Questionnaire in Iraqi refugees. Journal of immigrant and minority health. 2014; 16:1055-61.

5. Berthold SMMRFSDTAKLJLJ. The HTQ-5: revision of the Harvard Trauma Questionnaire for measuring torture, trauma and DSM-5 PTSD symptoms in refugee populations. European Journal of Public Health. 2019; 29:468-74.

6. Hollifield MWTDLNKBJJHKJSJWJ. Measuring trauma and health status in refugees: a critical review. JAMA JAMA. 2002; 288:611-21.

7. Tingh??g PMAACSELASF. Prevalence of mental ill health, traumas and postmigration stress among refugees from Syria resettled in Sweden after 2011: a population-based survey. BMJ Open BMJ open. 2017; 7:e018899.

8. Nabbe P, Le Reste JY, Guillou-Landreat M, Gatineau F, Le Floch B, Montier T, et al. The French version of the HSCL-25 has now been validated for use in primary care. PLOS ONE. 2019; 14:e0214804.

9. Oruc LKAPNMKFSMRFHDC. Screening for PTSD and depression in Bosnia and Herzegovina: validating the Harvard Trauma Questionnaire and the Hopkins Symptom Checklist. International Journal of Culture and Mental Health. 2008; 1:105-16.

10. Seabold S, & Perktold, J. , editor. statsmodels: Econometric and statistical modeling with python. In 9th Python in Science Conference; 2010.


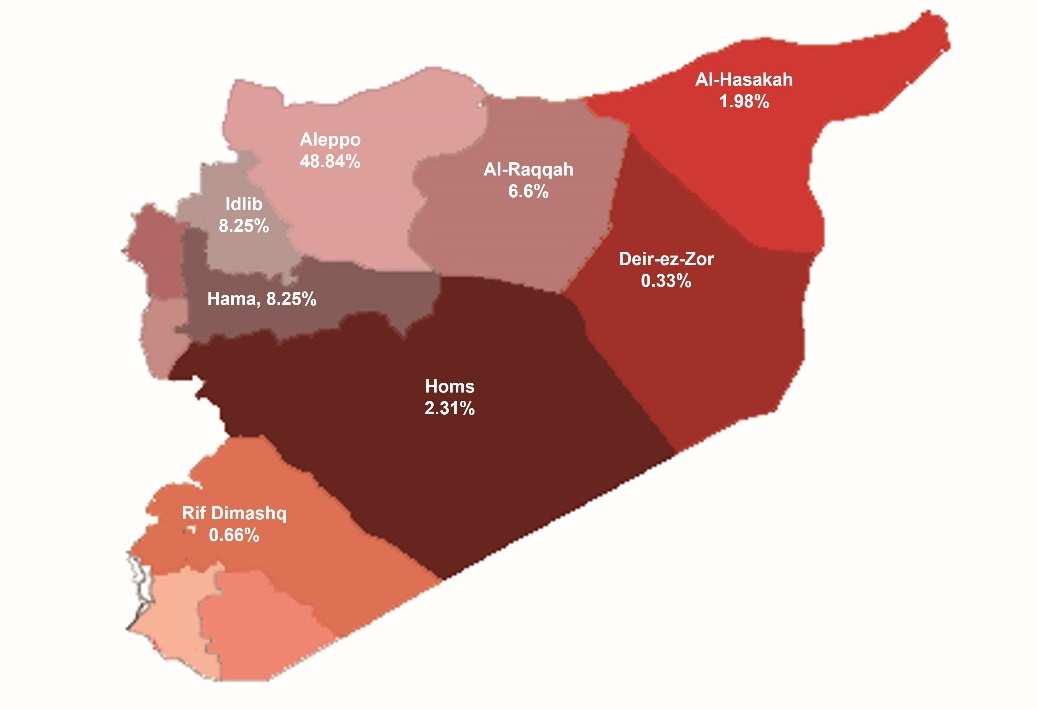


**Figure S1:** Geographical locations of the source region of Syrian refugees in the study sample


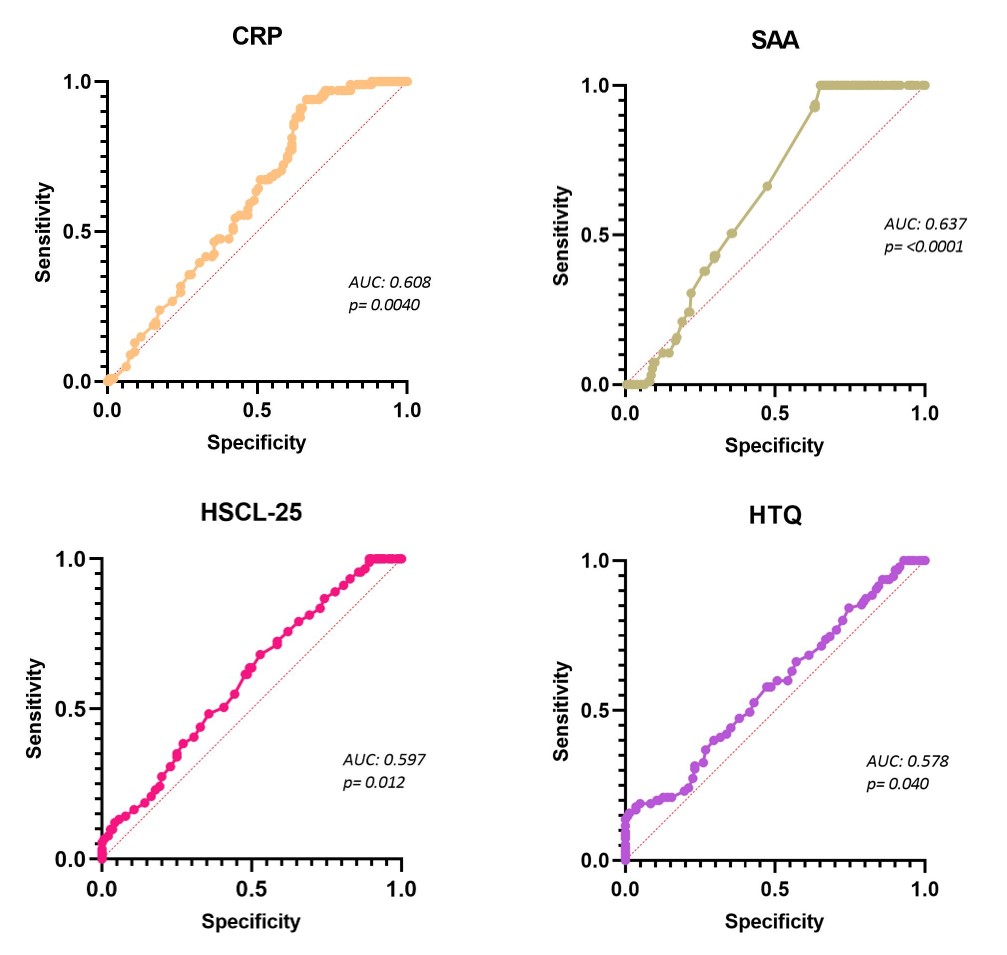


**Figure S2:** ROC curves of Inflammatory markers (SAA and CRP) and PTSD score (HTQ and HSCL-25) in predicting diabetes risk in the Syrian refugee population in Lebanon. Abbreviations: AUC = area under the ROC curve; ROC = receiver operating characteristic.


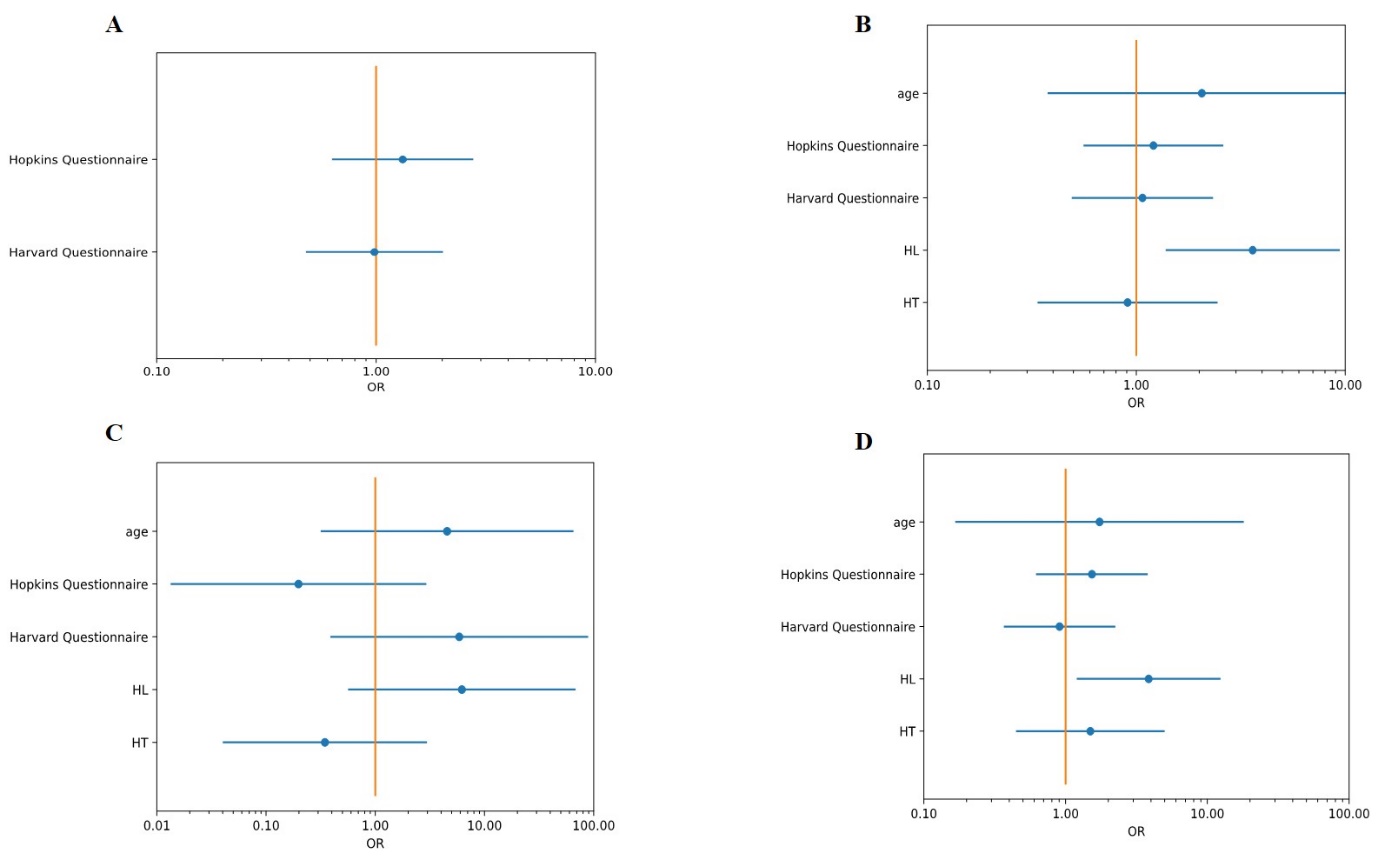


**Figure S3: (A)** Odds ratios of not previously diagnosed diabetic cases among refugees. Pseudo R-squared: 0.010, LLR p-value: 0.576 (**B)** Odds ratios of not previously diagnosed diabetic cases among refugees. Pseudo R-squared: 0.091, LLR p-value: 0.0622 **(C)** Odds ratios of not previously diagnosed male diabetic cases among refugees. Pseudo R-squared: 0.224, LLR p-value: 0.183 **D)** Odds ratios of not previously diagnosed female diabetic cases among refugees. Abbreviations: HL- Hyperlipidemia, HT- Hypertension. Pseudo R-squared: 0.103, LLR p-value: 0.138

| **Total (N=295)** | | **Normal**  (Total, Percentage) | **Prediabetic**  (Total, Percentage) | **Diabetic**  (Total, Percentage) |
| --- | --- | --- | --- | --- |
| **Men (95)** | <55 years (59) | 23, 7.79 | 14, 4.74 | 22, 7.46 |
|  | >55 years (36) | 12, 4.06 | 6, 2.01 | 18, 6.10 |
| **Women (200)** | <55 years (158) | 89, 44.5 | 33, 11.19 | 36, 12.2 |
|  | >55 years (42) | 10, 10.52 | 11, 3.72 | 21, 7.12 |

**Table S1:** Rates of diabetes mellitus risk factors stratified by gender and age.

|  | **Age** | **Weight** | **Height** | **BMI** | **Cholesterol** | **HDL** | **LDL** | **TG** | **Fasting Blood Sugar** | **HbA1c** | **CRP** | **HTQ** | **HSCL-25** |
| --- | --- | --- | --- | --- | --- | --- | --- | --- | --- | --- | --- | --- | --- |
| **SAA** | 0.001, 0.723 | 0.007, 0.208 | 0.001, 0.627 | 0.042, 0.003^*^ | 0.005, 0.339 | 0, 0.904 | 0.002, 0.551 | 0.002, 0.542 | 0.001, 0.628 | 0.003, 0.402 | 0.114, <0.0001^*^ | 0.003, 0.434 | 0.002, 0.476 |
| **HTQ** | 0.001, 0.571 | 0.001, 0.692 | 0.016, 0.075 | 0.013, 0.111 | 0.012, 0.104 | 0, 0.872 | 0.013, 0.123 | 0,  0.892 | 0.004, 0.38 | 0.005, 0.367 | 0.01, 0.12 | 0.003, 0.434 | 0.554, <0.0001^*^ |
| **HSCL-25** | 0,  0.784 | 0.001, 0.647 | 0.016, 0.073 | 0.006, 0.256 | 0.013, 0.086 | 0.002, 0.531 | 0.007, 0.239 | 0.001, 0.627 | 0,  0.994 | 0.007, 0.25 | 0.008, 0.17 | 0.002, 0.476 | 0.554, <0.0001^*^ |

**Table S2:** Pearson correlations between PTSD scores (HTQ, HSCL) and SAA levels against demographic and clinical characteristics of the study population. P<0.05 was considered significant. Each block reports R^2^, and p-value.

|  | **SE** | **OR** | **95% CI** | **P value** |
| --- | --- | --- | --- | --- |
| Age | 0.0121 | 1.093 | 1.068 to 1.120 | <0.0001^*^ |
| Gender | 0.257 | 0.7304 | 0.4385 to 1.204 | 0.2215 |
| Body Mass Index | 0.0266 | 1.141 | 1.085 to 1.205 | <0.0001^*^ |
| Smoking | 0.08485 | 0.9337 | 0.6227 to 0.9948 | 0.4189 |
| **Family history of metabolic diseases** |  |  |  |  |
| Type II Diabetes | 0.0201 | 0.9828 | 0.9273 to 1.020 | 0.3894 |
| Hyperlipidemia | 0.023 | 0.9768 | 0.9041 to 1.015 | 0.3068 |
| Hypertension | 0.0264 | 0.9712 | 0.8774 to 1.000 | 0.2695 |
| **Clinical Assessment** |  |  |  |  |
| Total Cholesterol | 0.0028 | 0.9974 | 0.9918 to 1.003 | 0.3473 |
| High-density lipoprotein (HDL) | 0.0102 | 0.9714 | 0.9515 to 0.9905 | 0.0044^*^ |
| Low-density lipoprotein LDL | 0.0035 | 0.9958 | 0.9888 to 1.003 | 0.2306 |
| Triglycerides (TG) | 0.002 | 1.004 | 1.000 to 1.008 | 0.0562 |
| Fasting blood sugar | 0.0093 | 1.066 | 1.048 to 1.087 | <0.0001^*^ |
| HbA1c | 0.9042 | 688.2 | 137.8 to 4854 | <0.0001^*^ |
| C-Reactive protein (CRP) | 0.2213 | 1.751 | 1.199 to 2.872 | 0.0114^*^ |
| Serum amyloid A (SAA) | 0.0128 | 1.015 | 0.9924 to 1.044 | 0.2324 |
| **PTSD assessment** |  |  |  |  |
| HTQ | 0.2799 | 0.8686 | 0.4994 to 1.503 | 0.6148 |
| HSCL-25 | 0.2319 | 0.8092 | 0.5116 to 1.275 | 0.3612 |

**Table S3:** Results for multiple logistic regression analysis to indicate Odds Ratios (OR) for diabetes prognosis in refugees against various predictor variables

| **Stress Score** | **OR** | **95CI-** | **95CI+** | **P-value** |
| --- | --- | --- | --- | --- |
| **HTQ** | 0.983 | 0.479 | 2.018 | 0.962 |
| **HSCL-25** | 1.322 | 0.630 | 2.776 | 0.460 |

**Table S4**: Logistic regression for Not previously Diagnosed Diabetic subjects against non-diabetics.

|  | **OR** | **95CI-** | **95CI+** | **P-value** |
| --- | --- | --- | --- | --- |
| **Hypertension (HT)** | 0.908 | 0.337 | 2.449 | 0.849 |
| **Hyperlipidemia (HL)** | 3.606 | 1.382 | 9.409 | 0.009 |
| **HTQ** | 1.071 | 0.491 | 2.332 | 0.864 |
| **HSCL-25** | 1.207 | 0.559 | 2.608 | 0.631 |
| **Age** | 2.059 | 0.377 | 11.251 | 0.405 |

**Table S5**: Logistic regression for Not previously Diagnosed Diabetic subjects against non-diabetics, including hypertension, Hyperlipidemia, and age. Fit parameters for males: Pseudo R-squared: 0.224, LLR p-value: 0.183; for females: Pseudo R-squared: 0.103, LLR p-value: 0.138.

|  | **Male** | | | | **Female** | | | |
| --- | --- | --- | --- | --- | --- | --- | --- | --- |
|  | **OR** | **95CI-** | **95CI+** | **P-value** | **OR** | **95CI-** | **95CI+** | **P-value** |
| **Hypertension (HT)** | 0.346 | 0.040 | 2.979 | 0.334 | 1.494 | 0.447 | 4.994 | 0.514 |
| **Hyperlipidemia (HL)** | 6.205 | 0.564 | 68.240 | 0.136 | 3.854 | 1.199 | 12.389 | 0.024 |
| **HTQ** | 5.870 | 0.388 | 88.865 | 0.202 | 0.906 | 0.366 | 2.241 | 0.831 |
| **HSCL-25** | 0.198 | 0.013 | 2.931 | 0.239 | 1.533 | 0.618 | 3.798 | 0.357 |
| **Age** | 4.544 | 0.316 | 65.270 | 0.265 | 1.734 | 0.166 | 18.090 | 0.645 |

**Table S6**: Logistic regression for Male and Female Not previously Diagnosed Diabetic subjects against non-diabetics, including hypertension, Hyperlipidemia, and age.
